# Angiogenesis

**DOI:** 10.1155/2015/135861

**Published:** 2015-03-29

**Authors:** Qiang Zhao, Zongjin Li

**Affiliations:** ^1^The Key Laboratory of Bioactive Materials of Ministry of Education, College of Life Sciences, Nankai University, Tianjin 300071, China; ^2^Tianjin Key Laboratory of Tumor Microenvironment and Neurovascular Regulation, Nankai University School of Medicine, Tianjin 300071, China

The development of new blood vessels, termed as angiogenesis, is essential to embryonic growth and throughout life for physiological repair processes. The field of angiogenesis research was established approximately in 1970s by the Folkman hypothesis that tumor growth is angiogenesis-dependent [1]. In addition to the tumor growth, another promising field where angiogenesis has drawn attention as a critical target is cardiovascular diseases [2]. Therefore, inhibiting angiogenesis is a promising strategy for treating diseases like cancer, while promoting angiogenesis may benefit the ischemic diseases such as myocardial infarction. Molecular insights into these processes and new therapeutic approaches are therefore particularly promising tools to understand the underlying pathologies and expand the available therapeutic options in abnormal angiogenesis. This special issue on angiogenesis highlights recent advances in our understanding of the molecular and cellular mechanisms of neovascularization. The summary of this special issue could be found in Figure 1.

With more bioassays and preclinical experiments performed, progress in understanding the relationship between tumor growth and angiogenesis has created a new perspective on therapeutic angiogenesis. Metformin is one of the most efficacious and safe front-line antidiabetics for type 2 diabetes (T2D). Gao et al. provided an overview of metformin as potential anticancer treatment by inhibiting angiogenesis. Tumor angiogenesis also influenced tumor microenvironment and C. Liu et al. described that exogenous overexpression of ATF4 (activating transcription factor 4) may facilitate the recruitment of macrophages into tumor tissues and promote tumor angiogenesis. Though atherosclerosis will trigger ischemia diseases, a clearer prescription of angiogenesis accounting for the development of the plaque of atherosclerosis is well recognized. F. Vasuri et al. evaluated the relationship between the intraplaque angiogenesis and the clinical plaque instability. Furthermore, L. Wang et al. reviewed the antiangiogenesis effects of polymethoxyflavones (PMFs), which underline the important of traditional medicine for anticancer therapy.

Ischemic cardiovascular diseases are the major cause of mortality and morbidity, which appeals for more effort on the better and more useful treatments. Therapeutic angiogenesis with stem cell and biomaterial has appeared as a powerful option. B. Chiara et al. described the angiogenic properties of mesenchymal stem cells (MSCs) and further emphasized the role of SIRT1. D. Mao et al. reported the therapeutic effects of SKP (skin-derived precursor cell) in stroke by promoting neurogenesis and angiogenesis. Furthermore, K. Wang et al. developed hybrid PCL/gelatin fibrous scaffolds with sustained release of VEGF, which could enhance vascularization* in vivo*. D. P. Zankov et al. reviewed the involvement of actin cytoskeleton-associated junctional molecules in angiogenesis and lymphangiogenesis, which may present a new approach to angiogenic therapy.

In general, this special issue on angiogenesis covered both excessive angiogenesis and insufficient angiogenesis (Figure 1). Either the reports or the reviews on angiogenesis presented in this issue will provide a further understanding about the real role of angiogenesis in cardiovascular diseases or cancer.

*Qiang Zhao* *Zongjin Li*

## Figures and Tables

**Figure 1:**
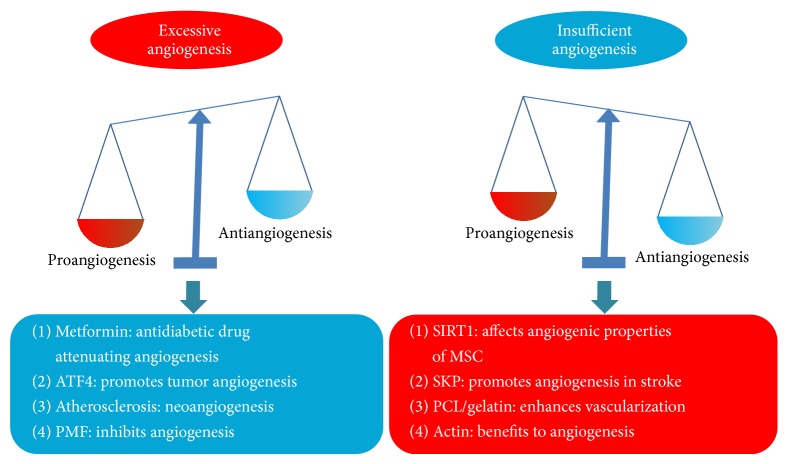
Summary of this special issue on angiogenesis. The impaired balance between pro and antiangiogenic factors causes pathological angiogenesis. Molecular insights described in this special issue provide new therapeutic strategies in abnormal angiogenesis management.
